# *In vivo* retinal imaging is associated with cognitive decline, blood-brain barrier disruption and neuroinflammation in type 2 diabetic mice

**DOI:** 10.3389/fendo.2023.1224418

**Published:** 2023-10-02

**Authors:** May Majimbi, Samuel McLenachan, Michael Nesbit, Fred K. Chen, Virginie Lam, John Mamo, Ryu Takechi

**Affiliations:** ^1^ Curtin Health Innovation Research Institute, Faculty of Health Sciences, Curtin University, Bentley, WA, Australia; ^2^ Lions Eye Institute Australia, Harry Perkins Institute of Medical Research, Nedlands, WA, Australia; ^3^ Perron Institute for Neurological and Translational Research, Nedlands, WA, Australia

**Keywords:** diabetic cognitive decline, blood brain barrier, correlation, neuroinflammation, oxidative stress, retina neurodegeneration

## Abstract

**Introduction:**

Type 2 diabetes (T2D) is associated with chronic inflammation and neurovascular changes that lead to functional impairment and atrophy in neural-derived tissue. A reduction in retinal thickness is an early indicator of diabetic retinopathy (DR), with progressive loss of neuroglia corresponding to DR severity. The brain undergoes similar pathophysiological events as the retina, which contribute to T2D-related cognitive decline.

**Methods:**

This study explored the relationship between retinal thinning and cognitive decline in the LepR *db/db* model of T2D. Diabetic db/db and non-diabetic db/+ mice aged 14 and 28 weeks underwent cognitive testing in short and long-term memory domains and *in vivo* retinal imaging using optical coherence tomography (OCT), followed by plasma metabolic measures and *ex vivo* quantification of neuroinflammation, oxidative stress and microvascular leakage.

**Results:**

At 28 weeks, mice exhibited retinal thinning in the ganglion cell complex and inner nuclear layer, concomitant with diabetic insulin resistance, memory deficits, increased expression of inflammation markers and cerebrovascular leakage. Interestingly, alterations in retinal thickness at both experimental timepoints were correlated with cognitive decline and elevated immune response in the brain and retina.

**Discussion:**

These results suggest that changes in retinal thickness quantified with *in vivo* OCT imaging may be an indicator of diabetic cognitive dysfunction and neuroinflammation.

## 1 Introduction

Type 2 diabetes (T2D) induces significant cognitive decline and is an independent risk factor for Alzheimer’s disease (1). Cognitive deficits in T2D encompass multiple domains, including spatial awareness, memory formation and executive function. These deficits can range from less-pronounced decrements to advanced impairment that resembles vascular and/or Alzheimer’s dementia pathologies (2). The underlying and perhaps heterogenic mechanisms are largely unknown; however, evidence in preclinical dietary-induced T2D models (3) and in genetic LepR db/db mice (4) suggests that cerebral microvascular disruptions, chronic neuroinflammation and brain atrophy appear as crucial pathogenic events in T2D-related cognitive deficits. Cerebral microvascular disruption describes compromised blood-brain barrier (BBB) function, which is associated with the activation of microglia in brain tissue and infiltration of circulating leukocytes (5, 6). In animal models of T2D, these neurovascular changes precede memory impairment assessed by spatial learning challenges (3, 4, 7).

Determining cerebral microvascular disruption and neuroinflammation early may support a better cognitive trajectory in individuals living with T2D. Hyperpermeability of the BBB and neuroinflammation have been occasionally reported in patients with T2D using advanced magnetic resonance imaging (MRI)-based techniques (8).

The retina is neural-derived, and several lines of evidence suggest it may serve as a surrogate marker of cerebrovascular integrity (9). In T2D, significant changes in retinal thickness measured non-invasively using optical coherence tomography (OCT) are reported (10). Moreover, cross-sectional and longitudinal studies have shown that retinal thinning in T2D is associated with impairment in global cognitive function (11, 12). However, these studies did not consider a putative association between retinal thickness and cerebrovascular integrity per se. Thus, the objective of this study was to investigate whether changes in retinal thickness measured by OCT are associated with disruption to the BBB, neuroinflammation and cognitive decline in a well-established clinically relevant mouse model of T2D.

## 2 Materials and methods

### 2.1 Animals

Male mice with spontaneous homozygous mutation in the leptin receptor gene (db/db) and heterozygote *db/+* of C57BLK/6J background were obtained from the Jackson Laboratory, the US and were maintained at the Animal Resource Centre, Western Australia. Animals at 4 weeks of age were group housed in a temperature-controlled laboratory at Curtin University on a 12 h light/dark cycle with standard chow (AIN93M, Specialty Feeds, WA) and water provided ad libitum. Following 7 d of acclimatisation, animals were randomly separated into 14-week and 28-week experimental endpoints. Body weight was measured prior to euthanasia. Experiments were conducted according to approved animal ethics protocol (Curtin Animal Ethics Committee, approval no. ARE 2018-19).

### 2.2 Cognitive assessment

#### 2.2.1 Novel object recognition

The protocol for novel object recognition was adapted from a previous study (13). The set-up consisted of an open field box (45 cm x 45 cm x 40 cm) situated in a dimly lit room with a video camera that enabled movement tracking and automated data collection using HVS Image 2014 software (HVS Image, UK). During the first day, mice were habituated to the arena for 10 min without any objects. On the second day, two identical objects were placed in the arena in opposite corners approximately 10 cm away from the arena wall. Mice were exposed to the objects for 10 min during familiarisation phase, then placed back in their home cage. Animals that spent less than 10 s with each object were excluded from the study. An object was selected at random and replaced with a different, novel object. After 2 h, mice were returned to the arena for 5 min during the test phase. Exploration in the familiarisation and test phases was defined as sniffing the object. The arena and objects were regularly sprayed with 70% (v/v) ethanol to minimise odor cues. The preference index (PI) was calculated as the time spent exploring the novel object in the test phase relative to the combined time spent exploring both objects, summarised as: Preference index (PI)= Novel object[s]/(Novel object[s] + familiar object[s] x 100.

#### 2.2.2 Passive avoidance

With a minimum of 24 h rest after the novel object recognition test, Passive Avoidance test was run according to a previous study (14) with several amendments, using step-through apparatus (Ugo Basile, Italy). Briefly, mice were placed in the illuminated chamber of the apparatus with the following experimental conditions for training phase: 1000 lux light intensity, 30 s door delay, 300 s step-through time, 2 s shock at 0.3 mA, and 10 s for the mouse to remain in the dark chamber prior to transfer to home cage. Animals that failed to enter the dark chamber during the training phase were excluded from the assessment. After 24 h, mice were returned to the illuminated chamber for the testing phase with identical experimental conditions, except the animals entered the dark chamber without a shock. Latency was calculated as the time taken to enter the dark chamber during the testing phase minus the time taken on training phase.

### 2.3 Optical coherence tomography

Retinal imaging was carried out according to a published protocol with minor alterations (15). Mice were anaesthetised using ketamine/xylazine (90 mg/kg ketamine, 12.5 mg/kg xylazine), and administered with eye drops containing 1% tropicamide and lignocaine (Akorn, Inc.) to dilate the pupils. Retinal imaging was conducted using an OCT imaging system (Heidelberg Engineering, Germany) set to a 30° field of view. Mice were placed into the holding compartment and fitted with a custom contact lens to prevent formation of temporary cataracts. Volume scans were obtained of the posterior pole of the eye from the optic nerve head (ONH) to the retinal periphery. B-scan cross-sectional images were stored and thickness of the: 1) total neuroretina, 2) ganglion cell complex (including the nerve fibre, retinal ganglion cell and inner plexiform layers), and 3) inner and outer nuclear layers were measured around the ONH (central) and 400 μm superior to the ONH (mid-peripheral) in nasal-temporal regions. Mean thicknesses of each layer were averaged from 6 measurement points across each B-scan using FIJI software (ImageJ, US).

### 2.4 Euthanasia and sample collection

Following OCT imaging, deeply anaesthetised mice were exsanguinated *via* cardiac puncture and euthanised by cervical dislocation. The brain and right eye were removed from the cranium and prepared as follows: the left hemisphere of the brain was snap-frozen in liquid nitrogen immediately after dissection and stored at -80°C, whereas the right hemisphere of the brain and right eye were fixed in 4% paraformaldehyde overnight and cryopreserved in 20% sucrose for 3 d. The right eye was transferred to 0.1% sodium azide and stored at 4°C and the right hemisphere was frozen in isopentane/dry ice slurry prior to storage at -80°C. To prepare 20 μm coronal sections that contain both cortex and hippocampus regions, brains were embedded in Optimal Cutting Temperature medium and cut on the Leica CM1520 Cryostat onto Poly-Lysine coated slides (Tranjan).

### 2.5 Plasma measures for glucose, triglycerides, and insulin resistance

Blood obtained in EDTA-coated syringes was centrifuged at 4°C for 10 min at 4000 rpm. The supernatant plasma was aliquoted and stored at −80°C for further analysis. Non-fasting plasma glucose was measured at an optimised dilution of 1:100 using a commercial colorimetric assay kit (Abcam). Insulin levels were assessed with the Ultrasensitive Mouse Insulin ELISA kit (Mercodia). Finally, plasma triglycerides were measured using a colorimetric assay (Randox). All plasma measures were analysed according to the manufacturer’s instructions. Insulin resistance was calculated using Homeostasis model assessment–insulin resistance (HOMA-IR) with the HOMA Calculator version 2.2.3 (Diabetes Trials Unit), and Triglyceride-glucose (TyG) index according to formula indicated below:


ln [triglycerides (mg/dL) × glucose (mg/dL)/2]

### 2.6 Brain immunofluorescence

BBB integrity was investigated using an established protocol for quantifying extravasated plasma macromolecule, Immunoglobulin G (IgG) in the brain parenchyma (16). As reported in the protocol, sections were co-labelled with the capillary basement membrane marker, laminin α4 to improve identification of the microvascular boundary. Briefly, 20 μm coronal sections of snap frozen left hemisphere were fixed with 4% paraformaldehyde for 10 min at 20°C and non-specific binding sites were blocked in 10% donkey serum (ThermoFisher) for 30 min at 20°C. The sections were washed in 0.1 M phosphate buffer saline (PBS) and incubated with goat anti-laminin α4 (1:200, RnD Systems) in an antibody signal enhancer solution (17) overnight at 4°C. Sections were washed in PBS and incubated with donkey anti-goat IgG AlexaFluor 555 followed by goat anti-mouse IgG AlexaFluor 647 mixed in PBS, with each secondary incubation lasting 2 h at 20°C.

Brain neuroinflammation, glial reactivity and oxidative stress were assessed as described (14). Briefly, 20 μm thick fixed cryosections of the right hemisphere were rehydrated in PBS, and non-specific binding sites were blocked in 10% donkey serum (ThermoFisher) for 30 min at 20°C. Sections were incubated overnight at 4°C with either rabbit anti-ionized calcium-binding protein 1 (1:200, Iba1; Novachem) for microglia or a combination of goat anti-glial fibrillary acidic protein (1:500, GFAP; Abcam) for astrocytes and mouse anti-8-Hydroxyguanosine (1:500, 8-OHdG (15A3); Abcam) for DNA oxidation. Thereafter, sections were incubated with either goat anti-rabbit IgG AlexaFluor 488 (1:500, ThermoFisher) or a combination of donkey anti-goat IgG AlexaFluor 555 (1:1000, ThermoFisher) and 1:500 donkey anti-Mouse IgG AlexaFluor 647 (ThermoFisher) for 2 h at 20°C. DAPI was used to counterstain the nuclei.

Immunostaining of the cortex and hippocampus were captured at 20x magnification on the Zeiss Axioscan Z1 slide scanner (Carl Zeiss, Germany). Images were processed offline using Zeiss Zen Desk software, with Intellesis trainable segmentation module for leakage analysis (16). Semi-quantitative analysis of protein expression was determined using voxel intensity per volume for each region of interest.

### 2.7 Retina immunofluorescence

Retina immunofluorescence staining and imaging were carried out according to an optimised protocol developed in-house. To summarise, retinas were enucleated from mouse eyes in a petri dish containing 1XPB then transferred to a 96 well-plate, and incubated with Tris-EDTA Buffer (10mM, pH 6.0), overnight at 37°C. Retinas were blocked and permeabilised in a buffer containing 0.2% Tween20, 2% Triton X-100, 0.2% bovine serum albumin in PBS for 1 h at 20°C with gentle shaking. Retinas were washed in PBS and co-stained for microglia (1:500, Iba1; Novachem) and astrocytes (1:500, GFAP; Abcam) (1:500), overnight at 4°C with gentle shaking. For secondary antibodies, retinas were incubated with donkey anti-goat IgG AlexaFluor 647 (1:1000, ThermoFisher) for 2 h at 20°C with gentle shaking, then washed with PBS and incubated with goat anti-rabbit IgG AlexaFluor 546 (1:500, ThermoFisher) under identical conditions. Retinas were mounted onto Poly-Lysine coated slides (Tranjan) with the vitreous side facing upwards.

Immunofluorescence-labelled retinal wholemounts were imaged at 20x magnification using the Dragonfly Confocal Microscope (Andor, Oxford Instruments). Fluorescence micrographs were captured with the following settings for each excitation wavelength at: Iba1 – 594nm, 42.5ms exposure, 24% laser intensity; GFAP – 685nm, 42.5ms exposure, 18% laser intensity. Images were processed in FIJI software (ImageJ, US) and immunofluorescence intensities were analysed semi-quantitatively using Zeiss Zen Desk software.

### 2.8 Statistical analysis

Statistical analyses were made in GraphPad Prism 9 (U.S.A). Data were expressed as mean ± SEM and in all calculations, significance was regarded at *P*< 0.05. Distribution of the data was assessed using D’Agostino–Pearson omnibus normality test. The parametric one-way analysis of variance (ANOVA) followed by Fisher’s least significant difference (LSD) for pairwise comparisons were used for normally distributed data. Pearson’s coefficient was used to analyse the associations between OCT outcomes and biological and cognitive measures.

## 3 Results

### 3.1 Db/db mice exhibited an obese phenotype

**Figure 1:**
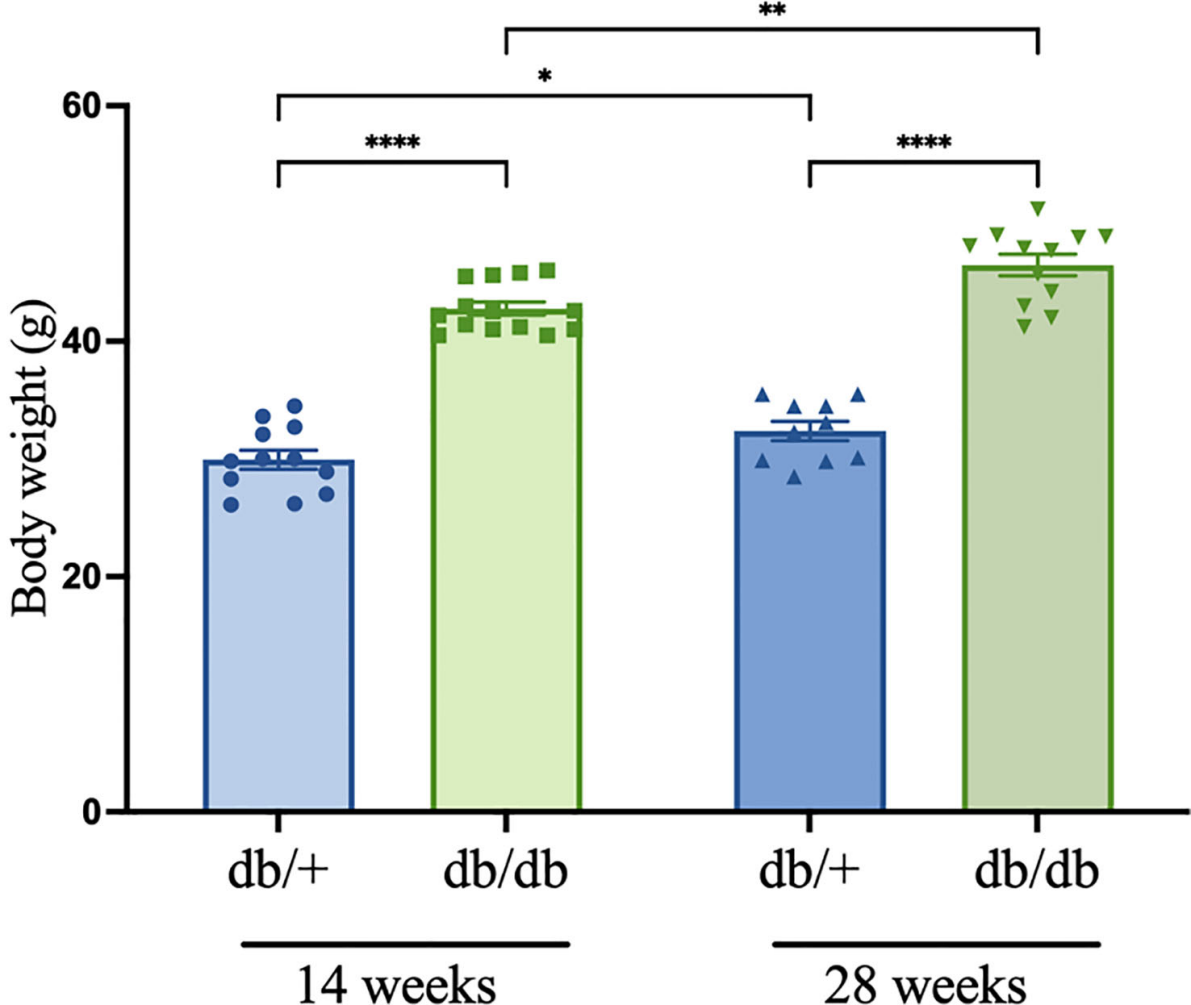
Body weight (g) measured prior to euthanasia in diabetic db/db and non-diabetic db/+ mice at 14 weeks and 28 weeks of age. Asterisks show statistical significance as *P<0.05, **P<0.01, ****P<0.0001. Animals weighed n = 8-12.

The T2D obese phenotype was evident in the db/db model (**Figure 1**). By 14 weeks of age, body weight of db/db mice was 40% higher compared to control db/+ mice. At 28 weeks, body weight had significantly increased in db/db mice by 57% and 19% compared to db/+ mice and 14-week db/db mice, respectively.

### 3.2 Db/db mice display hyperinsulinemia, hypertriglyceridemia, and diabetic insulin resistance

We measured non-fasting levels of plasma glucose, insulin, and triglycerides in diabetic db/db and non-diabetic db/+ mice. Subsequently, the extent of insulin resistance was assessed with calculations of HOMA-IR and TyG index (**Table 1**). Plasma glucose levels in db/db mice were approximately twice as high compared to age-matched control db/+ mice at 14 weeks and 28 weeks of age. There was a 30% difference in plasma glucose levels between db/db mice across the experimental groups. Diabetic hyperinsulinaemia was evident at 14 weeks of age, with significantly elevated insulin levels in db/db mice compared to db/+ mice. Furthermore, plasma insulin was significantly elevated in 28-week db/db mice compared to db/+ mice and 14-week db/db mice. Triglyceride levels were increased by 13% in 14-week db/db mice and 8% in 28-week db/db mice compared to their age-matched db/+ controls, however the results were not statistically significant. The results demonstrated insulin resistance with significantly elevated HOMA-IR in 14-week db/db mice compared to control db/+ mice. The elevation in HOMA-IR was greater in diabetic mice at 28 weeks of age, with a 500% increase relative to control db/+ mice. There was a significant 90% increase in HOMA-IR in diabetic db/db from 14 weeks of age to 28 weeks of age. Insulin resistance was confirmed with significantly elevated TyG index in db/db mice compared to age-matched db/+ mice at 14 weeks and 28 weeks of age.

**Table 1:** Blood glucose, plasma insulin, triglycerides, HOMA-IR and TyG index.

	14 weeks		28 weeks	
	db/+	db/db	db/+	db/db
Glucose (mg/dL)	275 ± 39.2	625 ± 61.8**	221 ± 36.4	438 ± 64.3**^,a^
Insulin (μg/L)	1.48 ± 0.139	2.71 ± 0.584*	2.47 ± 0.392	5.64 ± 0.208***^,a^
Triglycerides (mmol/L)	0.325 ± 0.045	0.275 ± 0.037	0.241 ± 0.032	0.364 ± 0.077
HOMA-IR	22.4 ± 3.52	78.8 ± 18.3*	19.8 ± 5.98	145 ± 23.6***^,a^
TyG index	6.63 ± 0.271	7.54 ± 0.339*	6.09 ± 0.138	7.32 ± 0.258**

HOMA-IR, Homeostatic Model Assessment Index—Insulin Resistance; TyG index, Triglyceride-glucose index. Values presented as Mean ± SEM. Asterisk indicates statistical significance compared to age-matched db/+ mice by post-hoc Fisher’s LSD test (*P<0.05, *P<0.05, **P<0.01, ***P<0.001). Superscript ^(a)^ notes statistical significance compared to 14-week db/db irrespective of degree of significance. Animals used for measures above n = 4-9.

### 3.3 Diabetes was linked to reduction in thickness of the inner retina

**Figure 2:**
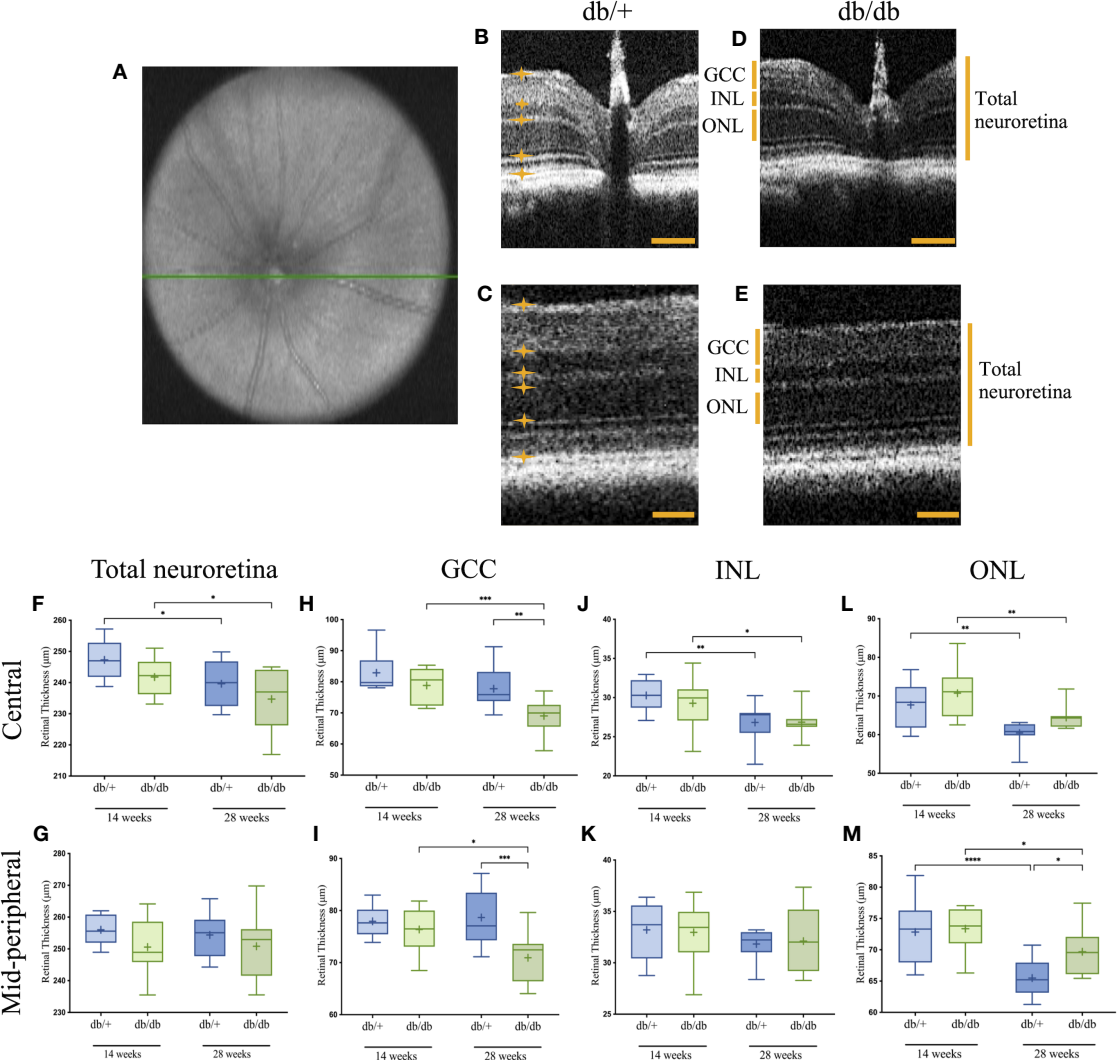
Representative OCT image of the central en-face scan with the optic nerve head indicated by a green line **(A)**, and B-scans of the central and mid-peripheral retinal regionals showing segmentation of the relevant layers in non-diabetic db/+ **(B, C)** and diabetic db/db **(D, E)** mice at 28 weeks of age. Six measurements were taken across each B-scan, with the boundary of each layer indicated by orange lines and asterisks. Scale bar is 50 µm. Boxplot graphs show data range with line through the median, and mean retinal thickness indicated as (+) for the GCC **(F, G)**, INL **(H, I)**, ONL **(J, K)**, and total neuroretina **(L, M)** in central (upper graphs). and mid-peripheral (lower graphs) regions with statistical significance noted as follows: *P<0.05, **P<0.01, ***P<0.001, ****P<0.0001. GCC, ganglion cell complex; INL, inner nuclear layer; ONL, outer nuclear layer. Animals used for OCT imaging analysis, with the right eye included in the analysis n = 7-12.

Retinal OCT imaging, as represented in **Figures 2A–E**, revealed both diabetes-associated and age-related thinning in the mice. The measures of total neuroretina thickness were comparable in the central and mid-peripheral retina between diabetic db/db and non-diabetic db/+ controls at 14 weeks and 28 weeks of age (**Figures 2F, G**). There was a significant age-dependent reduction of the total neuroretina in the central retinal region by over 7 µm (2.5%) in both diabetic db/db mice and non-diabetic db/+ controls.

Among the sublayers, the GCC in both central and mid-peripheral retinal regions was of similar thickness in diabetic db/db mice and aged-matched control db/+ mice at 14 weeks of age (**Figures 2H, I**). At 28 weeks, the mean GCC thickness in db/db mice was significantly reduced by an average of 8 µm (10%) compared with db/+ controls. Furthermore, the GCC in both central and mid-peripheral regions showed significant reduction by over 9 µm (7.5%) in diabetic db/db mice from 14 weeks to 28 weeks of age.

Measurement of INL thickness revealed comparable results between db/db and db/+ animals in both central and mid-peripheral retina at 14 weeks and 28 weeks of age (**Figures 2J, K**). Compared to 14-week non-diabetic db/+ and diabetic db/db mice, INL measurement in the central retina of 28-week db/db and db/+ mice was decreased by 2 µm (8%) and 3.5 µm (11%), respectively.

Similar to the INL, ONL thickness was comparable between db/db and db/+ mice at 14 weeks of age (**Figures 2L, M**). Significant age-related ONL thinning was observed in diabetic db/db mice by 6 µm (9%) and non-diabetic db/+ mice by 7 µm (10.5%) from 14 weeks of age to 28 weeks of age. However, the age-dependent ONL thinning was less evident in db/db mice, showing significantly thicker ONL at 28 weeks by an average of 4 µm (6%), compared to age-matched db/+ mice.

Taken together, T2D was linked to changes in retina ultrastructure across various layers; age-related changes were observed in the INL, ONL and total neuroretina. The most consistent finding from 2 sample regions of the retina was the decrease in thickness of the GCC with diabetes progression.

### 3.4 Diabetic mice exhibited cognitive decline in short- and long-term memory tests

**Figure 3:**
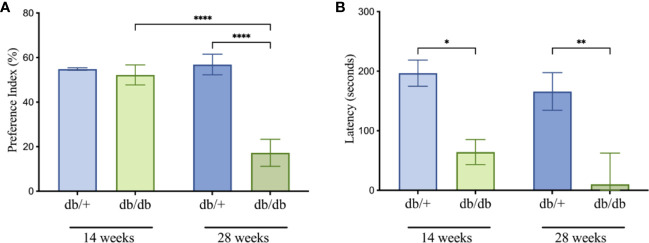
Cognitive testing for db/db and db/+ mice revealed deficits in the novel object recognition **(A)** and passive avoidance **(B)** tests. Statistical significance indicated as: *P<0.05, **P<0.01, and ****P<0.0001. Animals used for cognition measures n= 8-12.

To evaluate the impacts of diabetes on cognitive performance, mice underwent assessment of short-term and long-term memory using the novel object recognition (NOR) and passive avoidance (PA) tests, respectively (**Figure 3**). At 14 weeks of age, diabetic db/db mice exhibited intact short-term spatial learning and memory recall indicated by comparable Preference Index scores to non-diabetic control db/+ mice in the NOR test (**Figure 3A**). The Preference Index in db/db mice was significantly reduced compared to db/+ controls at 28 weeks of age, indicating substantial short-term memory deficits. The Preference Index was significantly lower in 28-week db/db mice compared to 14-week db/db mice, whilst the Preference Index in control db/+ mice showed no progressive deterioration of short-term memory.

In the PA test, diabetic db/db mice at 14 weeks of age showed significantly decreased latencies compared to age-matched non-diabetic db/+ mice, indicating long-term memory dysfunction (**Figure 3B**). The decrease in latency time was greater in 28-week db/db mice, also suggesting progressive loss of long-term memory.

### 3.5 Diabetic mice showed BBB breakdown

**Figure 4:**
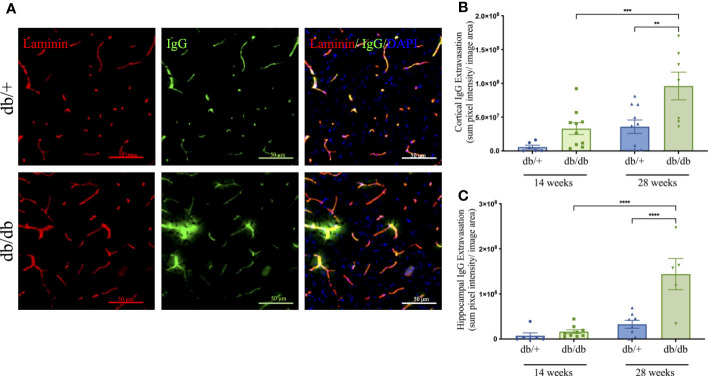
The blood-brain barrier integrity was assessed by immunostaining the basement membrane with laminin α4 (red) then detecting IgG extravasation (green) outside the capillary boundary, as shown in the representative images taken from the cerebral cortex of non-diabetic db/+ and diabetic db/db mice at 28 weeks of age **(A)**. Image scale bar is 50 μm. Mice at 14 weeks and 28 weeks of age were examined for parenchymal immunofluorescent IgG in the cerebral cortex **(B)** and hippocampus **(C)**. Statistical significance bars shown as **P<0.01, ***P<0.001, ****P<0.0001. Animals used for brain IgG analysis n= 6-10.

Cerebrovascular BBB integrity was examined using high throughput, automated quantitation of parenchymal extravasated plasma IgG (**Figures 4A–C**). At 14 weeks of age, db/db mice showed a non-significant 7-fold increase of IgG extravasation in the cerebral cortex compared to db/+ controls. Perivascular IgG extravasation in the hippocampus was comparable between db/db mice and db/+ controls 14 weeks of age. BBB dysfunction significantly increased with diabetic progression in db/db mice at 28 weeks of age, showing significantly elevated cortical and hippocampal IgG extravasation compared to age-matched db/+ controls and 14-week db/db mice (**Figures 4B, C**).

### 3.6 Diabetes induced heightened neuroinflammation and oxidative stress

**Figure 5:**
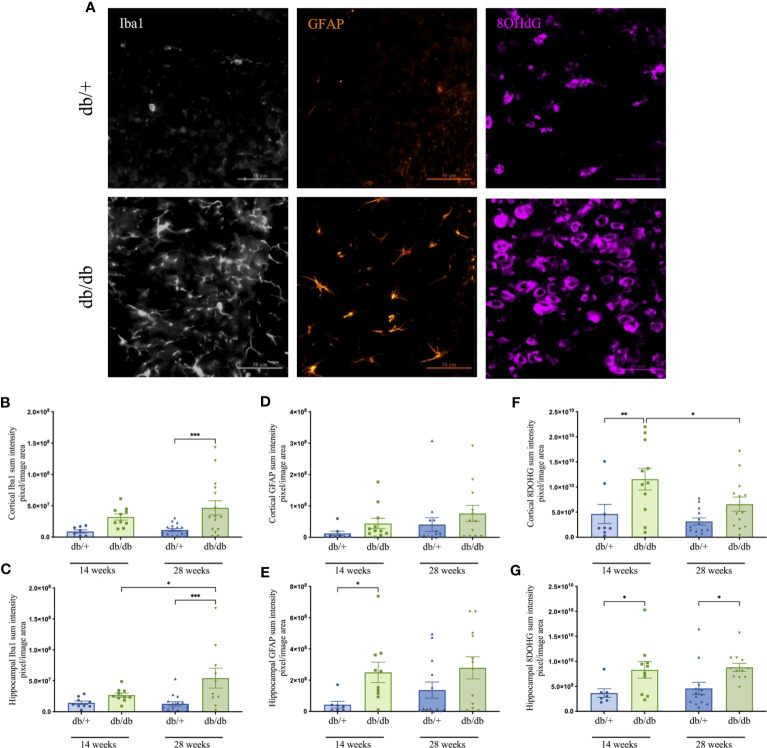
Representative images of Iba1- positive microglia in grey, GFAP- positive astrocytes in orange, and 8-OHdG cells that indicate DNA oxidation in violet captured within the cerebral cortex of non-diabetic db/+ and diabetic db/db mice at 28 weeks of age **(A)**. Image scale bar is 50 μm. Graphs show the immunointensities of Iba1 **(B, C)**, GFAP **(D, E)**, and 8-OHdG **(F, G)** in the cortex and hippocampus of db/db and db/+ mice at 14 weeks and 28 weeks. Statistical significance shown as *P<0.05, **P<0.01, ***P<0.001. Animals used for brain immunofluorescence measures n= 7-12.

Cerebral neuroinflammation and astrocytic activation were assessed *via* semi-quantitative intensity analyses of immunolabelled Iba1-positive microglia and GFAP-stained astrocytes, respectively. Oxidative DNA damage was evaluated using 8-OHdG immunoreactivity (**Figure 5**). Representative images are indicated in **Figure 5A**. At 14 weeks of age, db/db mice showed an average increase of 175% in Iba1 intensity compared to db/+ mice, although the difference did not reach significance (**Figures 5B, C**). There was a marked increase in microglial activation at 28 weeks, with significantly elevated Iba1 intensity confirmed in db/db mice relative to age-matched db/+ controls and the 14-week db/db group.

Reactive astrogliosis was prominent in the hippocampus of diabetic animals (**Figures 5D, E**). Indeed, db/db mice exhibited significantly elevated hippocampal GFAP intensity at 14 weeks compared to age-matched db/+ mice.

Quantitative analysis of 8-OHdG immunostaining revealed significant DNA oxidation in the cortex and hippocampus of 14-week db/db mice relative to db/+ mice (**Figures 5F, G**). Heightened oxidative stress persisted in db/db mice at 28 weeks with significantly greater 8-OHdG intensity in the hippocampus compared to db/+ mice. The 8-OHdG in the cortex of 28-week db/db mice was significantly reduced relative to 14-week db/db mice.

### 3.7 Diabetes increased retinal neuroinflammation

**Figure 6:**
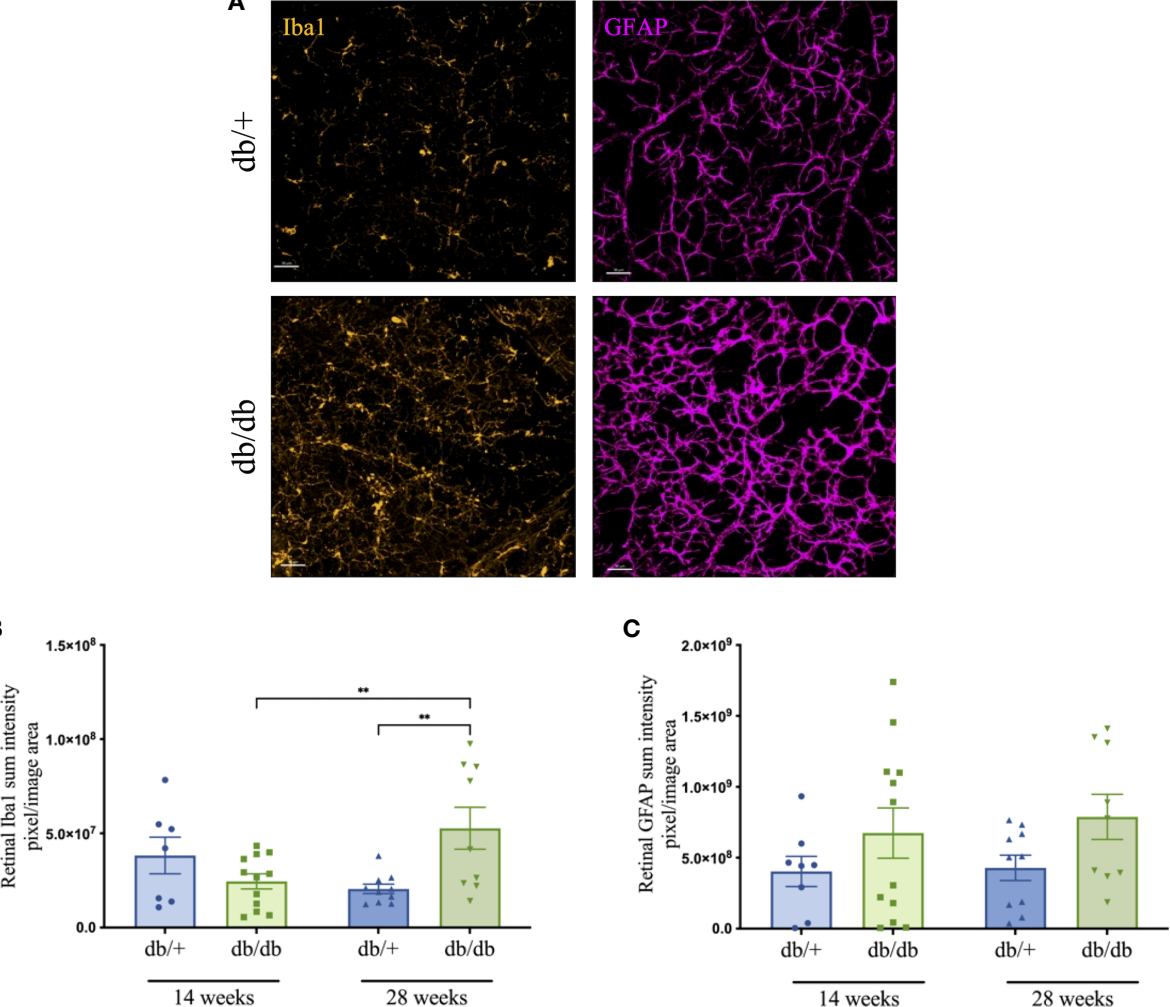
Representative immunofluorescence images (Iba1-positive microglia in orange and GFAP-positive astrocytes in violet) from the mid-peripheral region of retinal whole-mounts of non-diabetic db/+ and diabetic db/db mice at 28 weeks of age **(A)**. Scale bar is 50 μm. Changes in retinal Iba1 **(B)** and GFAP **(C)** immunointensity in db/+ and db/db mice at 14 weeks and 28 weeks of age. Statistical significance indicated as **P<0.01. Animals used for retinal immunofluorescence measures n= 8-12.

Microglial activation and astrocyte reactivity in the retina were examined *via* immunofluorescence microscopy, as shown in the representative images in **Figure 6A**. In 14-week db/db mice, Ibal immunointensity was statically comparable with db/+ controls. Microglial activation increased in db/db mice, showing significantly elevated Iba1 intensity in 28-week db/db mice compared to age-matched non-diabetic db/+ mice as well as 14-week db/db mice (**Figure 6B**). GFAP intensity in db/db mouse retina showed a modest 70% increase at both 14 and 28 weeks of age, compared to db/+ control mice (**Figure 6C**).

### 3.8 The retinal thinning in diabetes showed moderate correlations with cognitive decline disruption, neuroinflammation and oxidative stress

**Figure 7:**
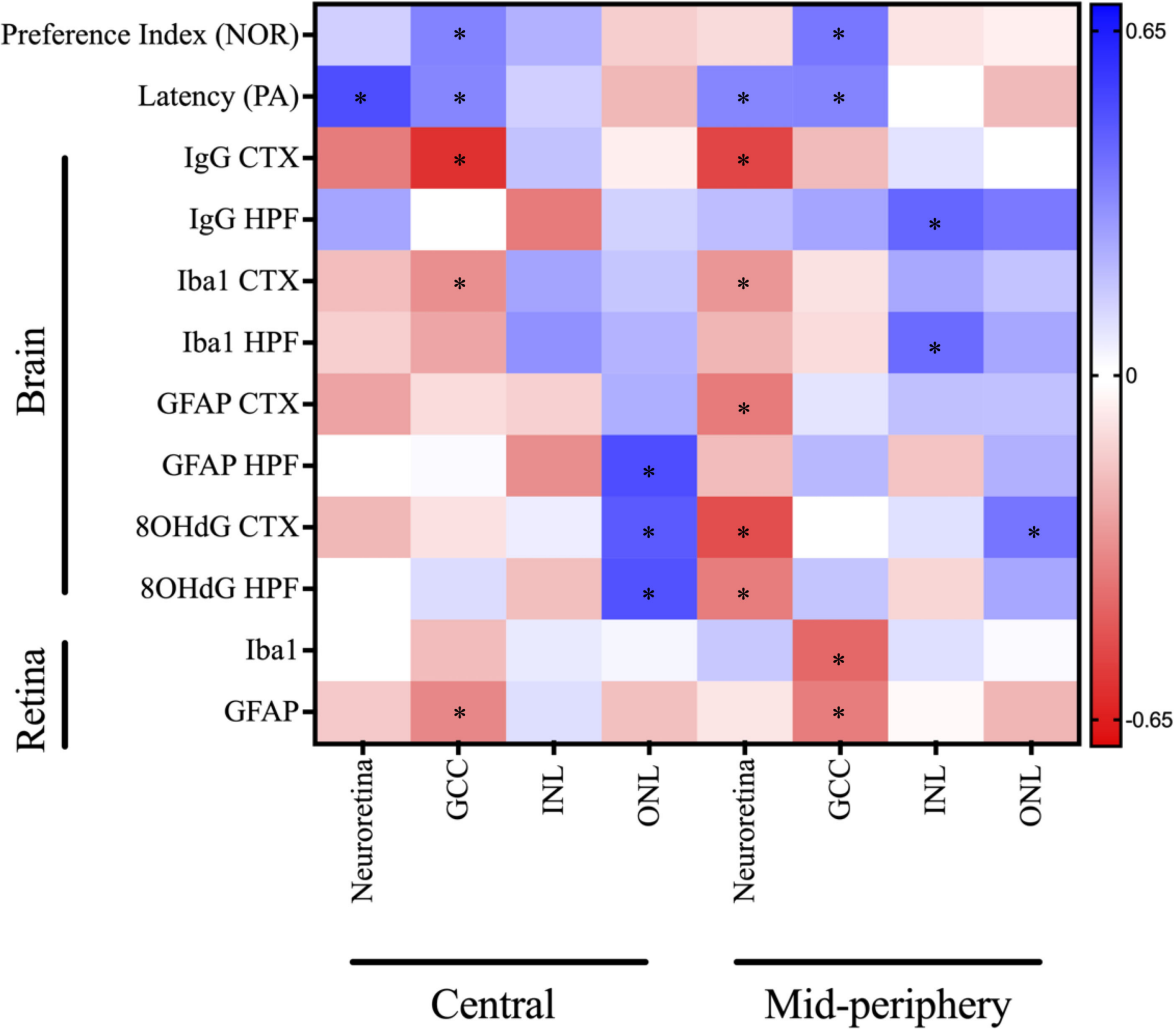
Correlation matrix of OCT measures against cognitive and immunofluorescence markers for neuroinflammation, oxidative stress, and BBB dysfunction depicted as a heatmap (corresponding colour scale provided and colour of each is cell is proportional to the value of the Pearson’s correlation coefficient). The notable correlations that either neared or reached statistical significance of *P<0.05 include: Neuroretina central: Latency (r = 0.50; P=0.003), GCC central: Preference Index (r = 0.35; P=0.06), GCC central: Latency (r = 0.34; P=0.05), GCC central: IgG CTX (r = -0.59; P=0.03), GCC central: Iba1 CTX (r = -0.32; P=0.12), GCC central: GFAP retina (r = -0.34; P=0.06), INL central: GFAP HPF (r = -0.33; P=0.15), ONL central: GFAP HPF (r = 0.50; P=0.02), ONL central: 8OHdG CTX (r = 0.47; P=0.02), ONL central: 8OHdG HPF (r = 0.49; P=0.02), Neuroretina mid-peripheral: Latency (r = 0.34; P=0.05), Neuroretina mid-peripheral: IgG CTX (r = -0.53; P=0.05), Neuroretina mid-peripheral: Iba1 CTX (r = -0.30; P=0.14), Neuroretina mid-peripheral: GFAP CTX (r = -0.38; P=0.07), Neuroretina mid-peripheral: 8OHdG CTX (r = -0.51; P=0.01), Neuroretina mid-peripheral: 8OHdG HPF (r = -0.37; P=0.01), GCC mid-peripheral: Preference Index (r = 0.38; P=0.04), GCC mid-peripheral: Latency (r = 0.35; P=0.05), GCC mid-peripheral: Iba1 retina (r = -0.43; P=0.01), GCC mid-peripheral: GFAP retina (r = -0.37; P=0.04), INL mid-peripheral: IgG HPF (r = 0.44; P=0.14), INL mid-peripheral: Iba1 HPF (r = 0.42; P=0.05), and ONL mid-peripheral: 8OHdG CTX (r = 0.39; P=0.06). CTX, cortex; GCC, ganglion cell complex; HPF, hippocampal formation; INL, inner nuclear layer; NOR, novel object recognition; ONL, outer nuclear layer; PA, passive avoidance.

Pearson’s correlation coefficient analysis revealed significant associations between retinal thickness and cognitive decline in diabetes (**Figure 7**). The total neuroretina thickness had a moderate positive correlation with performance in the PA test, but no apparent correlation with short-term recognition memory in the NOR test. Among the examined neural sublayers, GCC thinning in both central and mid-peripheral regions consistently correlated with short-term and long-term memory impairment in PA and NOR tests. In contrast, the relationship between INL thickness and cognitive performance varied from negligible to weak negative associations between INL (mid-peripheral) and Performance Index in the NOR test. There was a weak negative correlation between ONL thickness and cognitive outcomes.

Mixed results were found in the correlation assessment between retinal thickness and BBB permeability, as determined by IgG extravasation. The total neuroretina thickness in the central and mid-peripheral regions showed moderate negative correlations with IgG extravasation in the cortex; however, the relationship was reversed for the hippocampal formation. This trend was also observed in the sublayers, with GCC thickness in both regions of the retina negatively correlated with IgG extravasation only the cortex. There was a negligible to weak positive correlation between GCC thickness and IgG extravasation in the hippocampal formation. Conversely, INL thinning in the central, but not mid-peripheral, retina was linked to elevated IgG extravasation in the hippocampus. The ONL thickness at the central retina showed no correlation with global IgG extravasation, however, the mid-peripheral retina was positively correlated with hippocampal BBB permeability.

We also observed variable correlations between retinal thickness and neuroinflammation, measured as Iba1 intensity, in the cortex and hippocampal formation. The thickness of the total neuroretina and GCC showed weak to moderate negative correlations with Iba1 intensity, whereas the INL and ONL had weak to moderate positive correlations to neuroinflammation.

Furthermore, the total neuroretina thickness showed variable correlations to GFAP intensity, the principal measure of astrocyte reactivity. There was a moderate negative correlation with cortical GFAP, and a weaker negative correlation to hippocampal GFAP intensity. Within the retinal sublayers, the GCC thickness had negligible correlations with GFAP-positive astrocyte reactivity. Other notable correlations were between INL and ONL thickness in the central retina and GFAP intensity in the hippocampus, which showed contrasting moderate negative and positive associations, respectively.

Similarly, the marker of oxidative stress, 8OHdG in the cortex and hippocampus showed varied correlations with retinal thickness. The total neuroretina thickness in the mid-peripheral retina had a moderate negative correlation with cortical and hippocampal oxidative stress. In contrast, total neuroretina thickness in the central retina had weak negative and negligible associations to 8OHdG intensity in the cortex and hippocampus, respectively. The GCC and INL thickness had no correlation, whereas ONL thickness had a moderate positive correlation to oxidative stress.

Lastly, the relationships between retinal thickness and biomarkers of inflammation and gliosis were examined in the present study. The total neuroretina thickness showed no meaningful correlations with either retinal Iba1 or GFAP measures. In contrast, significant correlations were observed between GCC thinning and retinal neuroinflammation as well as astrocyte activation. Furthermore, the INL and ONL thickness showed weak correlations with Iba1-positive microglial and GFAP-labelled astrocytic activations, except for ONL (mid-peripheral) that had a weak negative relationship to retinal GFAP intensity.

Taken together, we observed that thinning in the total neuroretina and GCC was associated with elevated neuroinflammation, astrocyte reactivity, oxidative stress, and BBB permeability, apart from hippocampal IgG extravasation. Additionally, the total neuroretina and GCC layers showed the strongest relationship with memory performance in the PA and NOR tests. Conversely, ONL thickness had a reverse relationship to the total neuroretina and GCC pairwise comparisons.

## 4 Discussion

The metabolic sequalae of T2D, characterised by hyperglycaemia, insulin resistance, aberrant lipid metabolism and obesity, are evident in the db/db mouse model. As previously reported (18), diabetic mice in the present study exhibited a phenotype of obesity, elevated plasma glucose, a modest increase in plasma triglycerides, and decreased insulin sensitivity. These features of metabolic syndrome were present at 14 weeks of age and tended to worsen with diabetic duration. Specifically, the HOMA-IR and TyG index confirmed progressive insulin resistance in diabetic db/db mice at 28 weeks of age. Although HOMA-IR is widely utilised in clinical (19, 20) and preclinical diabetic literature (3, 21), TyG index is a relatively novel and inexpensive surrogate marker for insulin resistance that is positively correlated with HOMA-IR and glycated haemoglobin (HbA1c) (22, 23). Moreover, the TyG index shows promise in identifying individuals with T2DM-associated microvascular complications (24).

The present study investigated whether diabetic-induced changes in retinal thickness measured with *in vivo* OCT imaging co-occur with cognitive decline and its underlying pathophysiology, notably neuroinflammation, oxidative stress and BBB hyperpermeability measured by *ex vivo* immunomicroscopy. Our OCT analyses revealed that the thickness of the total neuroretina and sublayers GCC and INL were progressively reduced in diabetic mice. Our findings were consistent with previous reports of ganglion cell loss in humans (25) and preclinical models of T2D that include db/db mice (10, 26–28). Indeed, diabetes associated neurodegeneration is most often identified in the innermost retinal layers that comprise the GCC: 1) the retinal nerve fibre layer (RNFL) that contains axons of ganglion cells and forms the optic nerve, 2) the ganglion cell layer (GCL) that includes the cell bodies of retinal ganglion cells (RGCs), and 3) the inner plexiform layer (IPL) that houses the RGC dendrites and their synapses with other cells of the retina (10, 29, 30). The mechanisms underlying neurodegeneration are still under investigation; however, mounting evidence suggests that neurons in the GCC are highly susceptible to apoptosis under diabetic pro-inflammatory and oxidative stress conditions (27). The latter is consistent with our observations showing significant progressive increase of neuroinflammation and suggestive astrogliosis in the retina of db/db mice. Interestingly in the present study, ONL thickness was increased in diabetic db/db mice at 28 weeks of age. This finding is consistent with some reports that the nuclear layers increase in thickness with diabetes (30, 31).

Concomitant with the changes in retinal thickness, diabetic db/db mice exhibited cognitive decline, with impairments in cortical-hippocampal dependent short-term and hippocampal-amygdala mediated long-term memory tests (32, 33). Similar impairments have been published in preclinical diabetes literature (4, 34, 35) using Morris water maze (MWM) and Barnes maze as measures of spatial learning, memory and cognitive flexibility, and y-maze as a marker of working memory (33, 36). Cognitive decline in diabetes is supported by neuroimaging data that show altered activity in brain regions involved in cognition (8).

Whilst the underlying mechanisms by which diabetes induces cognitive decline are largely unknown, existing research indicates cerebral microvascular abnormalities are a critical pathophysiology of diabetic cognitive decline (35). The BBB is composed of non-fenestrated capillaries with closely joined endothelial cells (due to junction proteins), atop an extracellular matrix basement membrane interspersed with pericytes (37). Astrocytic end-feet encircle these microvessels. Additionally, these astrocytes project towards adjacent neurons, forming close synaptic connections. Microglia monitor the perivascular microenvironment where they mediate debris phagocytosis, detection of harmful stimuli and neuroprotection through release of anti-inflammatory markers during acute inflammatory states. Together, these cells constitute the neurovascular unit (NVU), and are crucial for maintaining appropriate neurological function (38). In a dietary-induced model of insulin resistance, the disruption of the BBB was demonstrated to causally associate with cognitive decline (3). BBB dysfunction is reported to chronically lead to heightened neuroinflammation and oxidative stress, resulting in neurodegeneration and cognitive decline (3, 21). Consistently, the current study showed progressive BBB hyperpermeability in diabetic db/db mice concomitant with neurocognitive decline. In parallel, significant neuroinflammation, indicated by microglial activation and astrogliosis, and elevated oxidative stress were observed in the hippocampal formation and cortex of db/db mice. The substantial neuroinflammation in the db/db retina was comparable to cerebral inflammation, suggesting that neural tissue dysfunction is linked to diabetic pro-inflammatory pathways.

Neurodegeneration, an inflammatory and oxidative stress-mediated process leading to neuronal loss through up-regulated apoptotic signalling, is central to the pathophysiology of T2D (39–41). Chronic activation of Iba1-microglia and GFAP-astrocytes observed in the present study leads to the release of pro-inflammatory cytokines, chemokines, and adhesion factors, exacerbating glial activation and attracting immune cells and macromolecules such as immunoglobulins from the plasma (42). In this context, reactive astrocytes no longer mediate cellular metabolism, synaptic glutamate clearance, and neurovascular coupling (43, 44). The latter pathogenic event reflects the essential role of astrocytes in autoregulation, which is defined as the adjustment of blood flow to meet energy demands. Neurovascular disruptions are reported in diabetes (38, 45) and neurodegenerative conditions where cognitive impairment is a major symptom (41, 46). Glial over-activation also triggers increased production of free radicals, toxic metabolites, nitric oxide (NO), and pro-angiogenic factors, impairing BBB integrity, leading to ischemic lesions and atrophy observed in T2D models and patients (47, 48). The subsequent neuronal and vascular loss disrupts neural plasticity and functional connectivity, essential cognitive mechanisms.

Diabetic-induced inflammatory and oxidative stress responses observed in the retina parallel those in the brain (49). While MRI scans show alterations in brain structure (8, 50), OCT imaging allows cost-effective examination of retinal neurodegeneration (51). Reductions in the inner retina thickness correspond to functional alterations detected with multifocal electroretinography (mfERG) (52), and damage to the superficial vascular plexus can be observed with OCT angiography (39), even in the absence of clinical microvascular pathology. Therefore, it is likely that any potential links between GCC thinning and memory impairment may be attributed to similar patterns of diabetic neurodegeneration in both tissues.

We examined the association between *in vivo* OCT measures of retinal thickness and short-term and long-term memory, as well as brain and retinal neuroinflammation, oxidative stress, and BBB dysfunction. Pearson’s correlation analysis revealed significant positive associations between retinal thickness and cognitive performance. In particular, thinning of the total neuroretina in the central and mid-peripheral regions was moderately correlated with impairment in long-term memory. Furthermore, GCC thickness in both retinal regions showed moderate positive associations with impairments in both short-term and long-term memory. Consistent with our findings, Allen et al. (53), reported that retinal thinning is concurrent with deficits in working memory and exploratory behaviour in the Goto-Kakizaki (GK) rat model of spontaneous T2D. Conversely, a thinner ONL layer was correlated with better cognitive performance, which is consistent with diabetic mice showing increased ONL thickness (30). The association between retina thickness and cognitive performance suggests that OCT imaging data may be used to identify elevated risk of cognitive decline in patients with T2D. This is supported by growing clinical evidence that confirms the link between diabetic retinopathy and cognitive decline, as reported in older people with T2D (54). The large-scale ACCORD trial by Hugenschmidt et al. (11), also reports cross-sectional and longitudinal correlations between retinopathy and diabetic cognitive decline, although changes in retinal thickness were not investigated.

The present data revealed significant associations between retinal thickness and cerebral BBB disruptions. Specifically, thinning of the total neuroretina and GCC in the central region were strongly associated with the cortical BBB disruption, whilst thinning of the INL was associated with elevated hippocampal BBB permeability. Upon examination of the mid-peripheral retina, there was an even stronger relationship between total neuroretina thinning and BBB disruption in the cortex. However, thickening of all retinal layers in the mid-peripheral region was associated with increased BBB permeability in the hippocampal formation. The underlying mechanisms for this regional variability remain obscure. Although IgG extravasation in the cortex and retinal ganglion cell layer has been shown to accompany retina thinning in diabetic pigs (55), no studies have yet correlated retinal structural changes to BBB hyperpermeability. Therefore, further research is necessary to explore the relationship between OCT retinal imaging and BBB integrity.

Our data also showed significant associations between changes in retinal thickness and brain pathophysiologies. The correlations between microglial activation and OCT retinal thickness were consistent across both retinal and brain regions, whereby total neuroretina and GCC thinning showed moderate associations with elevated microgliosis in the cortex; and the relationship was inversed for INL and ONL thickness. Astrogliosis and oxidative stress in the cortex and hippocampal formation were consistently associated with thinning of the total neuroretina in the mid-peripheral region and thickening of the ONL in both retinal regions. This suggests that structural changes across two or more retinal layers may be used in concert to improve the predictive capacity for cerebral pathophysiology. However more studies are required to replicate these findings and examine the temporal changes in the respective layers.

Finally, our correlation analysis revealed that GCC thinning was correlated with heightened neuroinflammation and astrogliosis in the retina, which confirms understanding that diabetic neurodegeneration is driven by chronic inflammation. To the best of our knowledge, this is the first study to investigate the relationship between retina thickness and a panel of neurophysiological markers for cognitive decline in a T2D model.

This study has some limitations due to the absence of repeated imaging and cognitive testing, which impedes the assessment of the predictive value of *in vivo* OCT imaging techniques. The implementation of a longitudinal study model would offer valuable insights into the temporal sequence of observed diabetic pathologies. Furthermore, pathological features of the db/db model such as hypertension were not explored in the study. Future studies should expand the battery of cognitive tests to evaluate deficits across various domains and their association with retinal microstructure. Furthermore, researchers should employ markers of neuronal loss to validate neurodegeneration *ex vivo* and corroborate OCT findings.

In summary, the results of the correlation assessment suggest that non-invasive *in vivo* OCT imaging may be utilised to identify a risk association with cognitive decline, cerebral BBB dysfunction and neuroinflammation in diabetes. These findings may be instrumental in the clinical detection of T2D patients at increased risk for future cognitive impairment, or who may already be exhibiting subtle, often overlooked, cognitive decrements The results also highlight the importance of considering specific retinal sublayers when investigating the relationship between the retina and cognitive function in T2D. However, further studies with more robust data sets are needed to increase the statistical power of the correlations.

## Data availability statement

The raw data supporting the conclusions of this article will be made available by the authors, without undue reservation.

## Ethics statement

The animal study was approved by Curtin Animal Ethics Committee, approval no. ARE 2018-19. The study was conducted in accordance with the local legislation and institutional requirements.

## Author contributions

MM - Responsible for day-to-day running of the animal model, coordinating and assisting with OCT imaging, conducting ex vivo measures and data analysis, writing and editing manuscript. SM – Animal anesthesia, collection of OCT images and guidance on interpreting OCT imaging data, editing manuscript. MN – Assistance with sample collection and optimisation of immunofluorescence staining and analysis. PB – Preliminary literature review on OCT. FC – consultation and advice regarding suitability of OCT imaging in animal model. VL –assistance with data interpretation, manuscript editing. JM – Guidance on animal model, writing and editing manuscript. RT – oversight of project, expertise on animal model and study design, assistance with data interpretation, writing and editing manuscript. All authors contributed to the article and approved the submitted version.
